# Effect of age as a continuous variable on survival outcomes and treatment selection in patients with extranodal nasal-type NK/T-cell lymphoma from the China Lymphoma Collaborative Group (CLCG)

**DOI:** 10.18632/aging.102331

**Published:** 2019-10-06

**Authors:** Wei-Xin Liu, Mei Shi, Hang Su, Ying Wang, Xia He, Li-Ming Xu, Zhi-Yong Yuan, Li-Ling Zhang, Gang Wu, Bao-Lin Qu, Li-Ting Qian, Xiao-Rong Hou, Fu-Quan Zhang, Yu-Jing Zhang, Yuan Zhu, Jian-Zhong Cao, Sheng-Min Lan, Jun-Xin Wu, Tao Wu, Su-Yu Zhu, Shu-Nan Qi, Yong Yang, Bo Chen, Ye-Xiong Li

**Affiliations:** 1 State Key Laboratory of Molecular Oncology and Department of Radiation Oncology, National Cancer Center, National Clinical Research Center for Cancer, Cancer Hospital, Chinese Academy of Medical Sciences (CAMS) and Peking Union Medical College (PUMC), Beijing, P. R. China; 2 Xijing Hospital, Fourth Military Medical University, Xi'an, Shaanxi, P. R. China; 3 307 Hospital, Academy of Military Medical Science, Beijing, P. R. China; 4 Chongqing Cancer Hospital and Cancer Institute, Chongqing, P. R. China; 5 Jiangsu Cancer Hospital and Jiangsu Institute of Cancer Research, Nanjing, P. R. China; 6 Tianjin Medical University Cancer Institute and Hospital, Key Laboratory of Cancer Prevention and Therapy, National Clinical Research Center for Cancer, Tianjin, P. R. China; 7 Union Hospital, Tongji Medical College, Huazhong University of Science and Technology, Wuhan, P. R. China; 8 The General Hospital of Chinese People's Liberation Army, Beijing, P. R. China; 9 The Affiliated Provincial Hospital of Anhui Medical University, Hefei, P. R. China; 10 Peking Union Medical College Hospital, Chinese Academy of Medical Sciences (CAMS) and Peking Union Medical College (PUMC), Beijing, P. R. China; 11 Sun Yat-sen University Cancer Center; State Key Laboratory of Oncology in South China; Collaborative Innovation Center for Cancer Medicine, Guangzhou, P. R. China; 12 Zhejiang Cancer Hospital, Hangzhou, P. R. China; 13 Shanxi Cancer Hospital and the Affiliated Cancer Hospital of Shanxi Medical University, Taiyuan, P. R. China; 14 Fujian Provincial Cancer Hospital, Fuzhou, Fujian, P. R. China; 15 Affiliated Hospital of Guizhou Medical University, Guizhou Cancer Hospital, Guiyang, P. R. China; 16 Hunan Cancer Hospital and the Affiliated Cancer Hospital of Xiangya School of Medicine, Changsha, P. R. China

**Keywords:** NK/T-cell lymphoma, age, prognosis, chemotherapy, radiotherapy

## Abstract

Purpose: The aim of this study was to determine the impact of analyzing age as a continuous variable on survival outcomes and treatment selection for extranodal nasal-type NK/T-cell lymphoma.

Results: The risk of mortality increased with increasing age, without an apparent cutoff point. Patients’ age, as a continuous variable, was independently associated with overall survival after adjustment for covariates. Older early-stage patients were more likely to receive radiotherapy only whereas young-adult advanced-stage patients tended to receive non-anthracycline-based chemotherapy. A decreased risk of mortality with radiotherapy versus chemotherapy only in early-stage patients (HR, 0.347, P < 0.001) or non-anthracycline-based versus anthracycline-based chemotherapy in early-stage (HR, 0.690, P = 0.001) and advanced-stage patients (HR, 0.678, P = 0.045) was maintained in patients of all ages.

Conclusions: These findings support making treatment decisions based on disease-related risk factors rather than dichotomized chronological age.

Patients and Methods: Data on 2640 patients with extranodal nasal-type NK/T-cell lymphoma from the China Lymphoma Collaborative Group database were analyzed retrospectively. Age as a continuous variable was entered into the Cox regression model using penalized spline analysis to determine the association of age with overall survival (OS) and treatment benefits.

## INTRODUCTION

Extranodal nasal-type NK/T-cell lymphoma (NKTCL) is rare but more prevalent in China than Western countries [1–3]. It is associated with Epstein-Barr virus infection, and frequently originates in the upper aerodigestive tract as early-stage disease in young men [1, 2]. Treatment outcomes for NKTCL have improved because of upfront radiotherapy (RT) utilization and effective chemotherapy (CT) [4–8].

Age at diagnosis is a prognostic factor and influences cancer treatment decisions [5, 9, 10]. Studies often dichotomize patients’ age at 60 years because 60 years is the median age for common lymphoma subtypes; dichotomizing also simplifies the statistical analyses, interpretation, and presentation of results [11–14]. Age >60 years has been incorporated into prognostic models for lymphomas, including diffuse large B-cell lymphoma (DLBCL) and NKTCL [5, 11, 15, 16]. However, most studies report a median age of 43–53 years for NKTCL, with only 14%–34% of patients older than 60 years [2, 3, 17–19]. The 60 year-old cutoff for NKTCL seems arbitrary, and extrapolations from DLBCL might not reflect patients’ clinical and biological heterogeneity [17, 20–22]. A considerable amount of prognostic information is lost by dichotomizing age (e.g., 15 year-old and 45 year-old patients assigned to the same age group [<60 years] differed significantly by prognosis and survival). It remains unclear whether age as a prognostic factor affects treatment selection [20–22]. Therefore, risk-stratified therapy by age dichotomization may lead to false-positive results and confound treatment decisions.

Interest in the effect of age as a continuous variable on the prognosis and treatment of NKTCL has increased since a linear pattern of increased mortality risk without an apparent age cutoff demarcating survival differences in thyroid and prostate cancers [9, 10, 23], and recent reports on accumulated gene aberrations with increasing age in DLBCL [24]. This study investigated the association of age as a continuous variable with survival outcomes in NKTCL patients, whether it was independent of tumor features and primary treatment, and its clinical value in making appropriate treatment decisions.

## RESULTS

### Age distribution and clinical characteristics

**Figure 1:**
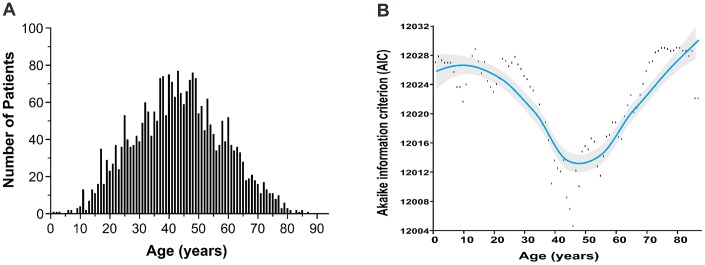
**Age distribution and optimal cutoff point by AIC analysis.** (**A**) age distribution follows the normal distribution with a median age of 43 years; (**B**) the optimal age cutoff value of 46 years was determined by AIC analysis. AIC, Akaike’s information criterion.

Age was normally distributed (median, 43 years; range, 1–87 years) (Figure 1A). A small proportion of patients was ≤21 (7.8%) or >60 years-old (13.8%) and the male to female ratio was 2.41:1 (Table 1). Most patients had early-stage disease (88.0%) and good performance status (PS), with Eastern Cooperative Oncology Group (ECOG) scores of 0–1 (91.6%); 31.8% had elevated lactate dehydrogenase (LDH), and 55.9% had primary tumor invasion (PTI).

**Table 1:** Patients’ characteristics and treatment by age group.

**Characteristic**	**All patients**		**Age groups (years)**						
			**≤ 21**		**22-45**		**46-60**		**> 60**
	**No. (%)**		**No. (%)**		**No. (%)**		**No. (%)**		**No. (%)**
Total	2640 (100)		206 (7.8)		1264 (47.9)		809 (30.6)		361 (13.7)
Male	1865 (70.6)		141 (68.4)		877 (69.4)		568 (70.2)		279 (77.3)
B symptoms	1059 (40.1)		107 (51.9)		538 (42.6)		305 (37.7)		109 (30.2)
ECOG 0-1	2418 (91.6)		185 (89.8)		1112 (92.3)		803 (92.5)		318 (88.1)
Elevated LDH	839 (31.8)		87 (42.2)		364 (28.8)		277 (34.2)		111 (30.7)
Nasal cavity	1957 (74.1)		145 (70.4)		924 (73.1)		621 (76.8)		267 (74.0)
Ann Arbor									
I	1579 (59.8)		93 (45.1)		746 (59.0)		512 (63.3)		228 (63.2)
II	746 (28.3)		74 (35.9)		372 (29.4)		207 (25.6)		93 (25.8)
III/IV	315 (12.0)		39 (19.0)		146 (11.6)		90 (11.1)		40 (11.1)
PTI	1475 (55.9)		122 (59.2)		731 (57.8)		428 (52.9)		194 (53.7)
Treatment									
CT alone	49.5 (18.8)		49 (23.8)		211 (16.7)		150 (18.5)		85 (23.5)
RT alone	393 (14.9)		21 (10.2)		153 (12.1)		130 (16.1)		89 (24.7)
CMT	1752 (66.4)		136 (66.0)		900 (71.2)		529 (65.4)		187 (51.8)

Abbreviations: ECOG: Eastern Cooperative Oncology Group; LDH: lactate dehydrogenase; PTI: primary tumor invasion; CT: chemotherapy; RT: radiotherapy; CMT: combined modality therapy.

### Linear-dependent effect of patients’ age on survival

**Figure 2:**
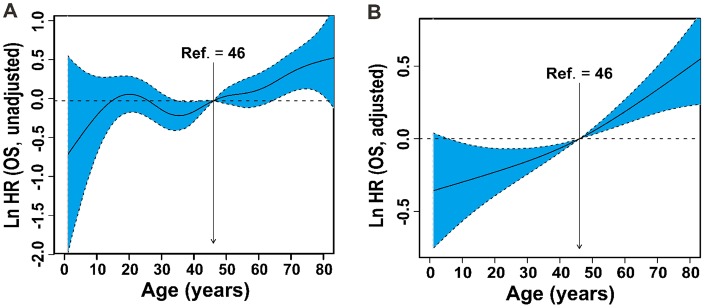
**Linear-dependent effect of increasing age on OS.** The estimated logarithm HRs (solid line) with 95% CIs (shading) for the association of patients’ age with OS in 2640 patients based on the the *dfmacox* in a smoothHR – the optimal extended Cox-type additive hazard regression unadjusted model (**A**) or the model adjusted for Ann Arbor stage, B symptoms, LDH, ECOG PS, and PTI (**B**). The effect of age on the risk of mortality was modeled using a penalized spline (*P*-spline) expansion, with patients’ age as a continuous covariate. An age cutoff of 46 years (indicated by the vertical line), defined by AIC analysis, was used as the reference value for calculating the HR. OS, overall survival; HR, hazard ratio; CI, confidence interval; *dfmacox,* degrees of freedom in multivariate additive Cox models; LDH, lactate dehydrogenase; ECOG, Eastern Cooperative Oncology Group; PS, performance status; PTI, primary tumor invasion; AIC, Akaike’s information criterion.

To quantify the prognostic effect, patient age as a continuous variable was entered into the Cox proportional hazards regression using *P*-splines in smoothHR to allow for a nonlinear relationship between age and OS. The risk (lnHR) of mortality increased steadily with increasing age (Figure 2A). After adjusting for covariates (PS, stage, LDH, PTI, and B symptoms), a similar trend in age and mortality was found (Figure 2B).

**Figure 3:**
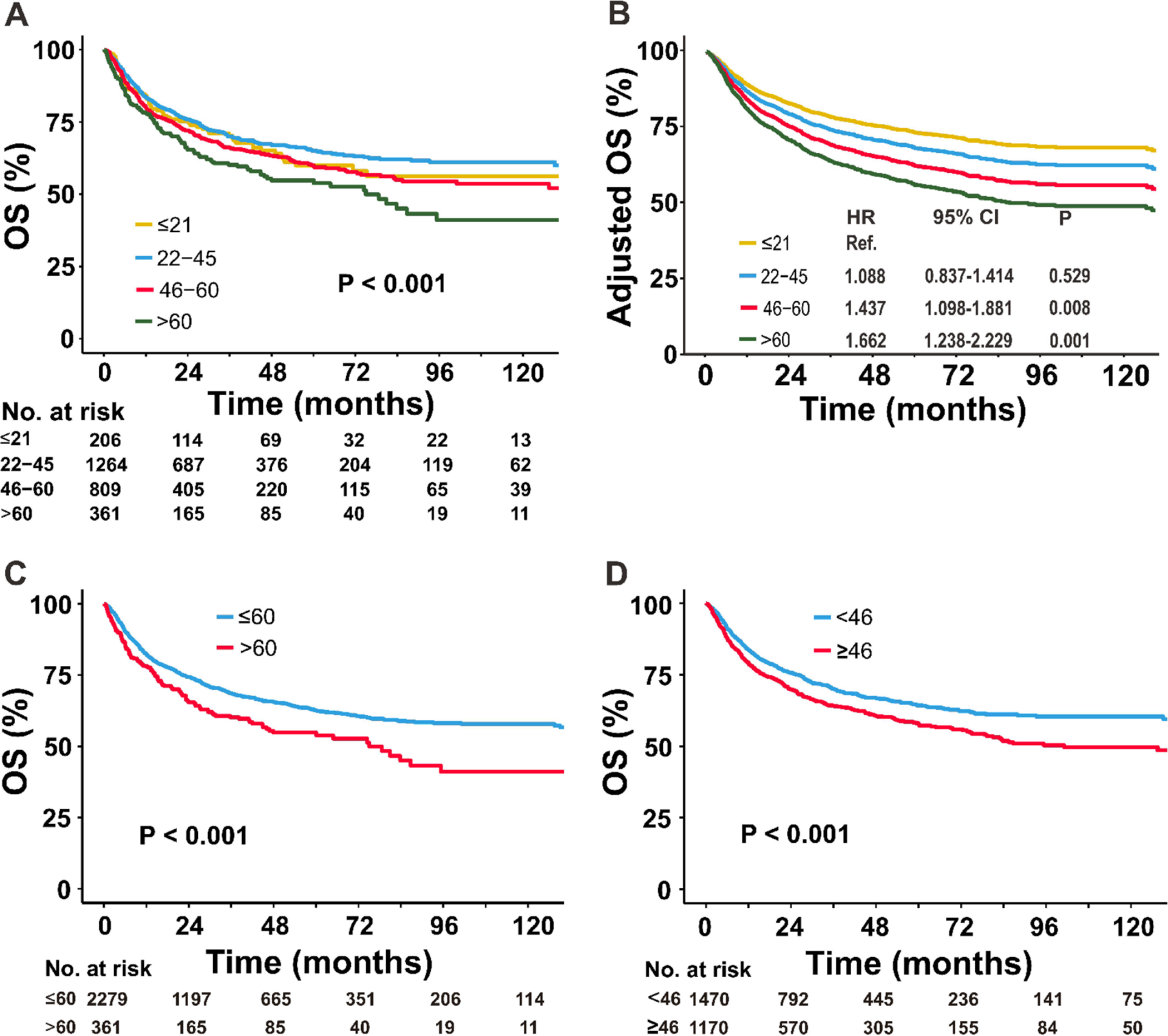
**Comparison of OS between the different age groups.** (**A**) Univariate and (**B**) multivariate analyses of the OS of patients stratified by age into four groups; (**C**) univariate analysis of the OS of patients stratified by age into two groups: ≤60 versus >60 years; and (**D**) <46 versus ≥46 years. OS, overall survival.

The optimal cutoff value of age was 46 years by AIC analysis (Figure 1B). Based on the AIC and commonly used cutoff points [15, 20, 21], patients were stratified into four age groups: ≤ 21 (children and adolescents), 22-45 (young-adult), 46-60 (adult) and > 60 years (elder). A comparison of survival differences showed children and adolescents were more likely than patients in the other groups to have adverse clinical factors, including B symptoms (51.9% vs. 30.2%–42.9%), elevated LDH (42.2% vs. 29.1%–33.4%), PTI (59.2% vs. 53.5%–57.7%), and advanced-stage disease (19.0% vs. 11.1%–11.7%) (Table 1). Before adjustment, patients ≤21 years-old had unadjusted OS comparable to those 46–60 years-old (Figure 3A). After controlling for all covariates, OS decreased with increased age (Figure 3B).

Significant differences in survival were observed between age groups after dichotomizing the data using a cutoff of 60 or 46 years. The unadjusted 5-year OS rates for the ≤60 versus >60 groups were 62.5% versus 54.8%, respectively (Figure 3C) and for the <46 versus ≥46 groups, the rates were 64.3% versus 57.8%, respectively (Figure 3D). After adjustment for all covariates, the adjusted OS remained significantly different between the two age groups (data not shown).

These findings indicated a positive linear relationship between increasing age and mortality without a cutoff point.

### Continuous variable of patients’ age as an independent prognostic factor for survival

**Figure 4:**
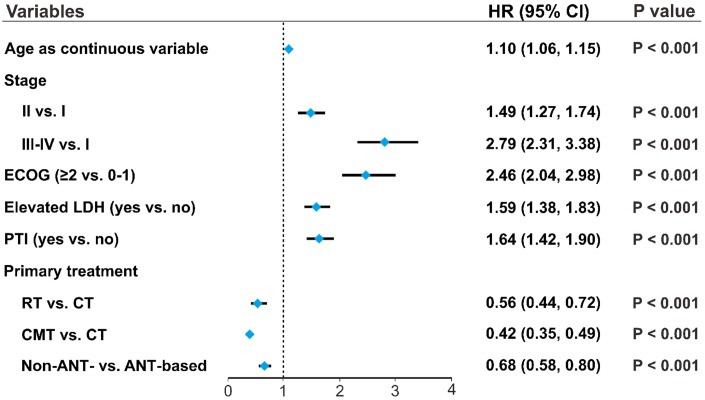
**Patients’ age as an independent prognostic factor.** Associations of clinical variables and primary treatment with OS were analyzed using multivariate analysis. Forest plots indicate the independent prognostic effects of patients’ age, as a continuous variable, and other clinical variables on OS. HRs were derived from multivariate Cox regression models, with 95% CIs and *P*-values for OS. OS, overall survival; HR, hazard ratio; CI, confidence interval.

Patients’ age as a continuous variable had a significant independent association with OS (HR, 1.10; 95% CI, 1.06–1.15) after adjusting for covariates and treatments (Figure 4). Other clinical factors, including stage, PS, LDH, and PTI significantly influenced OS. Compared with CT only, RT (HR, 0.56; 95% CI, 0.44–0.72) and CMT (HR, 0.42; 95% CI, 0.35–0.49) significantly decreased the risk of mortality. Non-ANT-based CT was associated with a significant improvement in OS compared with ANT-based CT (HR, 0.68; 95% CI, 0.58–0.80), indicating age, as a continuous variable, was an independent prognostic factor for OS, regardless of primary treatment.

### Impact of age on treatment selection

**Figure 5:**
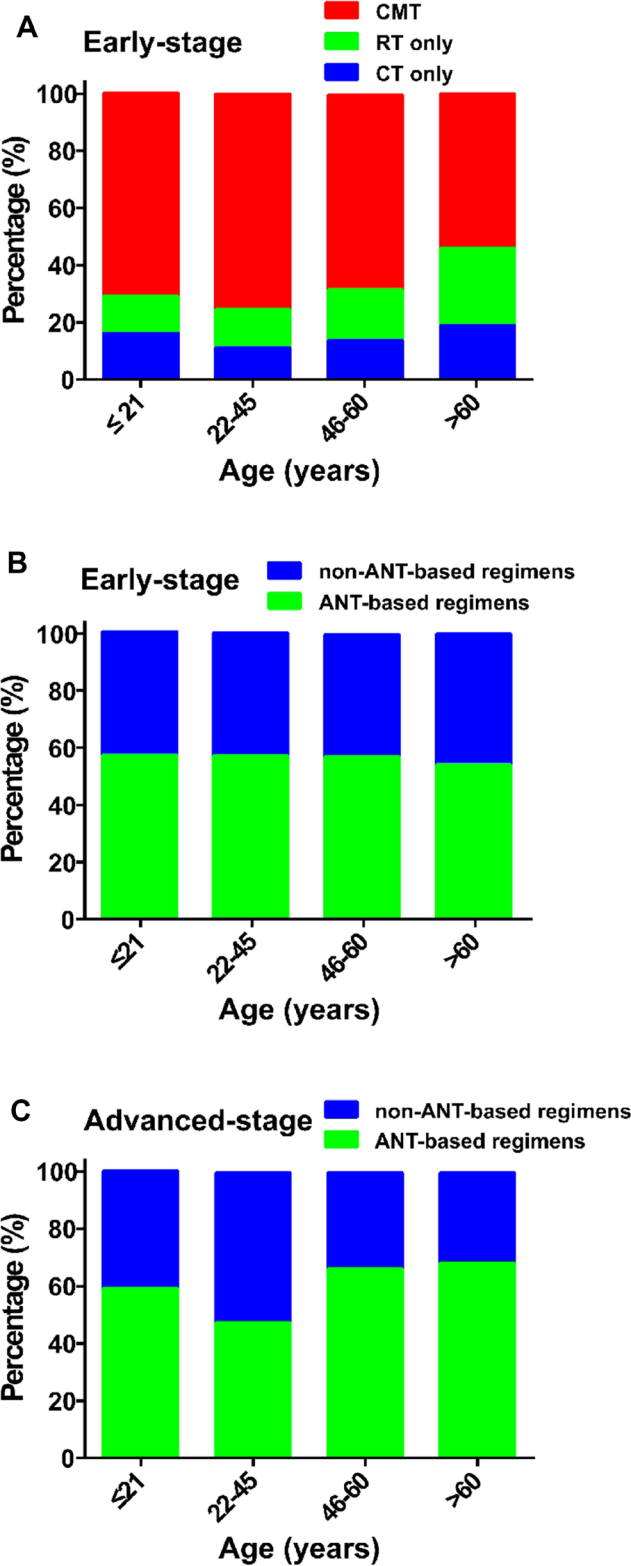
**Treatment strategies in various age groups.** (**A**) early-stage patients treated with CMT, RT or CT; (**B**) early-stage patients treated with a non-ANT-based or ANT-based regimens; and (**C**) advanced-stage patients treated with a non-ANT-based or ANT-based regimens. CMT, combined modality therapy; RT, radiotherapy; CT, chemotherapy; ANT, anthracycline.

Elderly early-stage patients were significantly more likely to receive RT only (24.7% age >60 versus 10.2% age ≤21; Figure 5A). No significant difference was found between non-ANT-based and ANT-based regimens in early-stage patients treated with CMT (Figure 5B). Young-adult, advanced-stage patients (22–45 years-old) tended to receive the non-ANT-based regimen, compared with the other age groups (65.5% versus approximately 50%, *P* < 0.05; Figure 5C).

### Constant survival benefits of RT in early-stage disease by age

**Figure 6:**
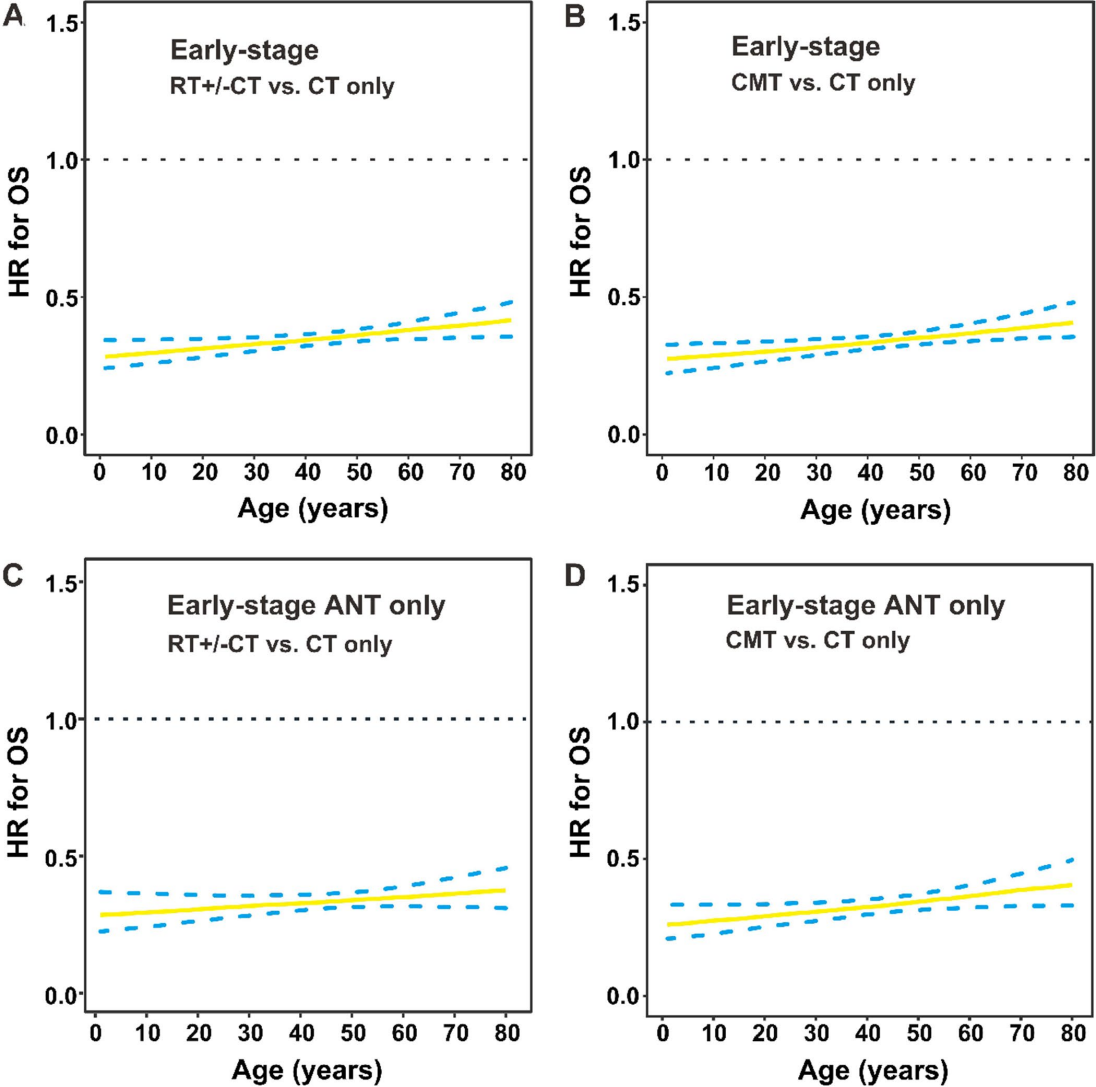
**OS by treatment modality and age group in early-stage patients.** (**A**) HRs for OS are presented by RT with or without CT versus CT only; (**B**) CMT versus CT only in early-stage patients; (**C**) RT with or without CT versus CT only in early-stage patients who received ANT-based regimens; (**D**) CMT versus CT only in early-stage patients who received ANT-based regimens. The solid line represents the HR estimate, and dashed lines represent 95% CIs. OS, overall survival; HR, hazard ratio; RT, radiotherapy; CT, chemotherapy; CMT, combined modality therapy; ANT, anthracycline.

The HR for OS after RT versus CT only was plotted when age was defined as a continuous variable in early-stage patients. Compared with CT only, RT (with or without CT) significantly decreased the risk of mortality in early-stage patients of all ages (HR for the entire group: 0.347, 95% CI, 0.287–0.420; Figure 6A). The performance of CMT compared with CT only also improved significantly among patients of all ages (HR, 0.339; 95% CI, 0.279–0.412; Figure 6B), indicating RT’s survival benefit was independent of age with early-stage disease. Similarly, for patients who received ANT-based CT, we also found a significant difference between RT+/-CT and CT only (HR, 0.333; 95% CI, 0.262–0.422; Figure 6C), CMT and CT only (HR, 0.328; 95% CI, 0.256–0.420; Figure 6D).

### Survival benefit of non-ANT-based CT by age

**Figure 7:**
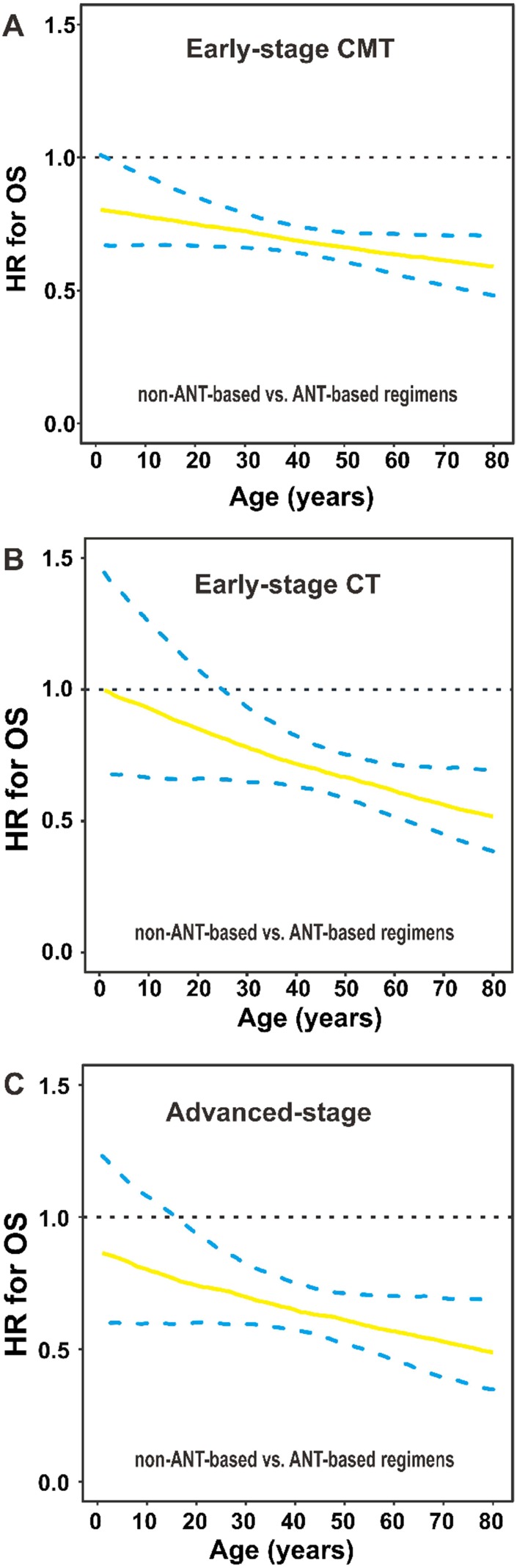
**OS by chemotherapy regimens and age group.** (**A**) HRs for OS are presented by non-ANT-based versus ANT-based regimens in early-stage patients who received (**A**) CMT or (**B**) CT, and (**C**) in advanced-stage patients. The solid line represents the HR estimate, and dashed lines represent 95% CIs. OS, overall survival; HR, hazard ratio; ANT, anthracycline; CMT, combined modality therapy; CT, chemotherapy; CI, confidence interval.

For early-stage patients receiving CMT, non-ANT-based regimen significantly decreased the risk of mortality compared with ANT-based regimen (HR for the entire group: 0.690; 95% CI, 0.553–0.860; Figure 7A). Furthermore, for early-stage patients receiving CT, there was a marginally significant difference between non-ANT-based and ANT-based regimens (HR for the entire group: 0.694; 95% CI, 0.476–1.013; Figure 7B). For advanced-stage patients, non-ANT-based CT significantly improved OS compared with ANT-based CT (HR for the entire group: 0.678; 95% CI, 0.463–0.992; Figure 7C). The relative performance of the non-ANT-based regimen compared with the ANT-based regimen improved with increasing age. This finding indicated the survival benefit of the non-ANT-based regimen was independent of age.

## DISCUSSION

This study found a linear relationship between older age at diagnosis and a higher risk of death after adjustment for clinical characteristics and primary treatment. No apparent age cutoff corresponded to a significant decrement in OS, challenging the appropriateness of age as a binary variable in prognostic analyses or current risk models for NKTCL [2, 16]. Treatment strategies varied between age groups, with elderly patients more likely to receive RT and less likely to receive non-ANT-based CT. RT for patients with early-stage disease and non-ANT-based CT for all stages provided a survival benefit for patients of almost all ages, indicating optimal treatment should depend on disease-related risk factors rather than chronological age.

NKTCL affects young males and a small proportion of children and older adults. By dichotomizing age, children and adolescents (≤21 years) had a favorable prognosis [20], unlike elderly patients (>60 years) [17, 21]. In recent prognostic models of NKTCL [2, 16], patients’ age is dichotomized with a cutoff of ≥60 years as an unfavorable risk factor. This age cutoff for NKTCL was derived from other non-Hodgkin lymphoma without considering their different age distributions and heterogeneity of molecular and clinical features [11–16]. This study is the first to reveal that age, as a continuous variable, is an independent prognostic factor without an apparent cutoff value. Consequently, stratification by two (at 46 or >60 years) or four age groups was sufficient for differentiating prognoses with significant differences in OS for NKTCL. This finding supports continuous age shifts in the risk of mortality from NKTCL and challenges current clinical concepts regarding prognostic stratification and treatment guidelines based on strict age cutoffs. Incorporating patients’ age as a continuous variable into prognostic or predictive modeling may contribute to improvements in risk stratification and better informed treatment decisions [25]. Further studies are needed to clarify genetic heterogeneity with increasing age and its impact on the prognosis of patients with NKTCL [26].

It is well known that older patients are offered less aggressive treatment than their younger counterparts, which might reflect overreliance on chronological age as a proxy for other risk factors, which may, or may not, be present. We confirmed that patients’ age influenced treatment decision making: older patients with early-stage disease were more likely to receive RT but less likely to receive a new CT regimen, whereas younger patients with early-stage disease were less likely to receive RT. As RT and non-ANT-based CT proved to be effective in treating patients with NKTCL [3–8, 27–30], the under-utilization of effective treatment for such patients is a clinical concern. Patients’ age, as a continuous variable, was an independent prognostic factor, but might not be a contraindication of curative RT and effective CT. The beneficial effect of RT or a non-ANT-based regimen was found among almost all patients, indicating no apparent cutoff age guiding treatment. Non-ANT-based regimens have been suggested as a first-line treatment for localized and advanced NKTCL because of the resistance to ANT-based regimens. However, it should be careful to recommend that elderly patients receive aggressive treatment. Based on these results, patients with NKTCL should utilize curative RT or explore innovative, effective systemic therapy in the clinical setting, regardless of their biological age [29], although many prospective trials exclude older and younger patients [7, 8].

This study has limitations related to its retrospective design. First, geriatric assessment data were unavailable. The adverse effect of non-ANT-based regimens in elderly patients and interactions of age-associated co-morbidities and treatments could not be analyzed. Second, the study was conducted from an endemic area using the CLCG database. Therefore, it is unclear whether these findings are generalizable to patients from non-endemic areas, such as Western countries [3, 19]. Third, assessments of an optimal risk model and chemotherapy regimen were not conducted because they were beyond this study’s scope.

In conclusion, this study was unique in its assessment of the prognostic effect of age as a continuous variable, and provided evidence for constant survival benefits of RT and non-ANT-based regimen in patients of all ages. These findings will be useful for oncologists in selecting the most appropriate treatments and designing prospective trials for patients with NKTCL.

## MATERIALS AND METHODS

### Study population

This retrospective study included 2640 previously untreated NKTCL patients with clinicopathologic factors from the China Lymphoma Collaborative Group (CLCG) database in 2000–2015. Diagnostic criteria and clinical evaluations have been described previously [5, 6]. Tumor cells were positive for NK/T-cell markers (CD3ε, CD56), cytotoxic molecules (T-cell intracellular antigen-1, granzyme B, perforin), and in situ hybridization for Epstein-Barr virus-encoded RNA, but negative for B-cell markers (CD20, CD79α). Our institutional review board approved the study, which was conducted in accordance with the Declaration of Helsinki.

### Treatment

Treatment options depended on patients’ Ann Arbor stages. Early-stage patients received CT only (*n* = 310), RT only (*n* = 387), or combined modality treatment (CMT; *n* = 1628), whereas advanced-stage patients received primary CT (*n* = 315). RT included the extended involved-site field at a median dose of 50 Gy [6]. Half of the patients received anthracycline (ANT)-based CT, and the other half received non-ANT-based CT. The median number of CT cycles was 4 (range, 1–20).

### Statistical analysis

The primary endpoint was overall survival (OS; measured from the beginning of treatment until the time of death from any cause or until the last follow-up). The chi-square test was used to compare treatment patterns between age groups. Survival was calculated using the Kaplan-Meier method, and OS differences between groups were compared using the log-rank test. Cox proportional hazards regression was performed to identify independent risk factors for OS. Akaike’s information criterion (AIC) model was used to determine the optimal age cutoff indicating survival differences. A multivariate Cox proportional hazards regression model with penalized spline (*P*-spline) was used to examine the relationship between age and OS [31]. *P*-spline provides a flexible model to examine the relationship between age and the natural logarithm of a hazard ratio (HR) without prior knowledge of the type of association, while adjusting for the effects of covariates. Performance of the treatment modalities was plotted by age using hazard ratios (HR) and 95% confidence intervals (CI) [32]. Statistical analyses were performed using SPSS 19.0, and the smoothHR, simPH, survminer, and maxstat packages in R, version 3.2.3 (http://www.r-project.org/).
